# Limitations of Aneuploidy and Anomaly Detection in the Obese Patient

**DOI:** 10.3390/jcm3030795

**Published:** 2014-07-17

**Authors:** Paula Zozzaro-Smith, Lisa M. Gray, Stephen J. Bacak, Loralei L. Thornburg

**Affiliations:** Department of Obstetrics and Gynecology, University of Rochester, 601 Elmwood Avenue, Box 668, Rochester, NY 14642, USA; E-Mails: paula_zozzaro-smith@urmc.rochester.edu (P.Z.-S.); lisa_gray@urmc.rochester.edu (L.M.G.); stephen_bacak@urmc.rochester.edu (S.J.B.)

**Keywords:** obesity, aneuploidy, ultrasound, anomaly, non-invasive prenatal testing, anatomy

## Abstract

Obesity is a worldwide epidemic and can have a profound effect on pregnancy risks. Obese patients tend to be older and are at increased risk for structural fetal anomalies and aneuploidy, making screening options critically important for these women. Failure rates for first-trimester nuchal translucency (NT) screening increase with obesity, while the ability to detect soft-markers declines, limiting ultrasound-based screening options. Obesity also decreases the chances of completing the anatomy survey and increases the residual risk of undetected anomalies. Additionally, non-invasive prenatal testing (NIPT) is less likely to provide an informative result in obese patients. Understanding the limitations and diagnostic accuracy of aneuploidy and anomaly screening in obese patients can help guide clinicians in counseling patients on the screening options.

## 1. Introduction

Within the past two decades, the prevalence of obesity has steadily increased worldwide. The most widely used measure for determining the prevalence of obesity is the body mass index (BMI), defined as the weight to height proportion expressed in kg/m^2^. The original classification system outlined by the World Health Organization in 1995 defined a BMI of 18.5–24.9 as normal weight, overweight as 25–29.9 and obese as 30 or greater [1]. This classification scheme was refined in 2000 to further categorize obesity into Class I/mild (30–34.9), Class II/moderate (35–35.9) and Class III/severe (>40) [2]. Results from the latest National Health and Nutrition Examination Survey (NHANES) found that 35% of adults in America are obese [3]. More than a third of childbearing-aged women in the United States are classified as obese, with the highest incidence seen among non-Hispanic black women (48%) compared with Hispanic women (43%) and non-Hispanic white women (33%) [3]. Maternal obesity increases the risk of pregnancy complications, including spontaneous abortion and congenital anomalies [4,5,6]. While obesity alone is not a risk factor for fetal aneuploidy, the prevalence of obesity increases with advancing age, which is clearly associated with aneuploidy risk. As the prevalence of maternal obesity in the reproductive age group continues to increase, prenatal screening options are of critical importance in this group of patients. Understanding the limitations of aneuploidy screening and prenatal ultrasound can assist clinicians in effective counseling and management of pregnancy in this population.

### 1.1. Worldwide Scope of the Obesity Problem

Obesity is a worldwide epidemic. Since 2000, it has been estimated that over 300 million adults worldwide are obese [7]. Obesity is now replacing malnutrition and infectious disease as a major cause of ill health within many developing countries [8]. A primary reason for the rapid rise in obesity has been the changing cultural and economic environment in both developed and developing countries, resulting in lower physical activity and an increase in the availability of high fat and high caloric foods [9]. In most countries, obese adults make up at least 20% of the population [9]. According to the Organization for Economic Co-operation and Developments (OECD), within the next 5–10 years, nearly two thirds of the population in OECD countries will be overweight or obese [10]. An Australian analysis of “the global burden of disease” found that, for women, more than 4% of total disability and life years lost was attributed to excess weight and obesity [11].

In addition to the complications of obesity, more women are entering pregnancy with obesity-related chronic conditions [12]. According to the most recent NHANES of more than 13,000 men and women, the prevalence of hypertension, dyslipidemia, metabolic syndrome and diabetes substantially increases with increasing BMI [13]. A review from the Framingham Heart Study noted that up to 30% of overweight and obese women had associated hypertension [14]. Furthermore, excessive weight substantially increases the risk for the development of type 2 diabetes [15].

### 1.2. Implications of Maternal Obesity and Weight Gain in Pregnancy

Obesity during pregnancy is a source of morbidity and mortality for both mother and the fetus. Obstetric risks of obesity include preeclampsia and other hypertensive disorders, gestational diabetes and cesarean delivery [6]. Women may gain excessive weight at any age, but the childbearing years are a time of particular risk. Additionally, more women enter pregnancy with obesity-related chronic conditions [12]. In 2009, the Institute of Medicine (IOM) revised guidelines for healthy ranges of weight gain in pregnancy. It is recommended that women in the normal BMI category gain 25–35 pounds, overweight women 15–25 pounds and obese women 11–20 pounds [12]. According to the Pregnancy Risk Assessment Monitoring System, the majority of women had excessive weight gain inconsistent with IOM recommendations, with a high percentage of women gaining more than 40 pounds during their pregnancies [12]. In one retrospective study of 53,000 women, the mean weight at first prenatal visit has progressively increased from 144 pounds in 1980 to 172 pounds in 1999 [16]. The percentage of women greater than 300 pounds at their first prenatal visit also increased ten-fold from less than 1% in 1980 to 5% in 1999 [16]. In one study of 902 postpartum women, 12% retained at least 11 pounds at one year postpartum [17]. Excessive pregnancy weight gain, as well as long-term weight retention are predictive of being overweight or obese later in life [18].

The obesity related co-morbid condition of pregestational diabetes mellitus has been shown to increase the overall risk of fetal anomalies by two-fold to four-fold [19]. While women with good glycemic control appear to have little to no increased risk above that in the general population, substantial hyperglycemia during organogenesis raises the risk of anomalies proportional to maternal glucose levels [19,20]. Such women are at a high risk of fetal sacral agenesis/caudal regression syndrome, neural tube defects and congenital heart defects [19,21,22]. Obesity alone appears to increase the risk of congenital anomalies; while such a risk may be related to undiagnosed diabetes, several studies have demonstrated consistent associations between maternal obesity and fetal malformations. In a meta-analysis by Stothard *et al.*, obese mothers were more likely to have a pregnancy affected by neural tube defects (OR 1.87, 95% CI 1.62–2.15), cardiac defects (OR 1.30, 95% CI 1.12–1.51), orofacial clefts (OR 1.20, 95% CI 1.03–1.40), anorectal atresia (OR 1.48, 95% CI 1.12–1.97), hydrocephalus (OR 1.68, 95% CI 1.19–2.36) and limb-reduction anomalies (OR 1.34, 95% CI 1.03–1.73) [21]. Blomberg *et al.* demonstrated similar findings among obese mothers in a population cohort of over 1,000,000 infants [5]. Additionally, their data demonstrated a dose-response relationship with the risks of defects increasing with the degree of obesity [5]. 

## 2. Aneuploidy Detection

While obesity alone is not a risk factor for fetal aneuploidy, obesity rates increase with advancing age, which is clearly associated with aneuploidy risk [23]. Additionally, maternal obesity has a negative impact on the diagnostic abilities of routinely used genetic screening tools. Patients should be informed of these limitations and made aware of the failure rates and the possible need for repeat testing or an altered testing strategy.

### 2.1. Nuchal Translucency Screening

The nuchal translucency (NT) screening is now routinely employed in the detection of fetal aneuploidy. Alone or in combination with first/second-trimester serum biochemical testing, NT screening has detection rates for trisomy 21 ranging from 71%–78% at a 5% false positive rate [24]. It is obvious that an inability to obtain an NT measurement precludes aneuploidy risk assessment using this method, although serum screening alone can still be used at the expense of lower detection rates [25]. While there are no prospective studies evaluating the performance of combined NT and first-trimester biochemical testing specifically in obese patients, several retrospective studies have examined the effect of obesity on the ability to obtain an NT measurement. Increasing BMI is associated with failure to obtain an adequate NT, longer ultrasound time and an increased need for transvaginal or repeat sonography [26,27]. Thornburg *et al.* showed that the rate of nuchal translucency screening failure increases with the degree of obesity, with a first attempt Class III obesity failure rate ten-fold higher than in non-obese patients (23% *vs.* 2.2%) [27]. With repeat attempts, the overall failure rate decreased to 6.6% (1.6% in non-obese), but remained strongly correlated with the degree of obesity (Class I 3.9%, Class II 6.7%, Class III 13.5%) [27]. Time to complete the ultrasound examination was also correlated with obesity with paradoxically shorter times noted in Class III obese patients. The authors postulated that these data may be due to increased rates of scan abandonment in such patients due to sonographer difficulty [27]. Gandhi *et al.* found that obese patients were four times as likely to have an inadequate nasal bone assessment (3.0% *vs.* 12.7%) and twice as likely to require transvaginal sonography (22% *vs.* 42%) [26]. In addition, patients with Class III obesity required 25% more time to complete the ultrasound examination (16 min *vs.* 20 min) [26]. While completion rates did not differ for obese patients in this study, the authors reported a 100% completion rate for all patients, which represents a substantial difference from other observational studies of NT measurements (58%–77%) [26,28]. Given these findings, obese patients should be counseled on the higher rates of screen failure and increased time required to complete their examination. Regardless, first-trimester screening should be used whenever possible, given increased sensitivity and specificity over second-trimester serum biochemical screening [29].

### 2.2. Serum Biochemical Screening

Maternal serum alpha-fetoprotein (AFP) has been used as a biochemical marker in open neural tube defect screening since the 1970s, with detection rates approaching 90% [30]. Several maternal serum biochemical markers have also been used for aneuploidy screening in the first or second trimester of pregnancy. Levels of human chorionic gonadotropin (hCG) and plasma-associated pregnancy protein-A (PAPP-A) are combined with NT measurements in the first trimester to provide risk assessments for trisomy 21 and trisomy 18 [31]. First-trimester screening has detection rates of 80%–87% for trisomy 21 (5% false-positive rate) and 75% for trisomy 18 (0.3% false-positive rate) [24]. Similarly, hCG, unconjugated estriol (uE3), alpha-fetoprotein (AFP) and dimeric inhibin-A (DIA) are measured in the four-analyte screen used in the second trimester for the detection of trisomy 18 (71%–100% detection rate at a 0.3% false-positive rate) and trisomy 21 (67%–71% detection rate at a 5% false-positive rate) [24,32]. When using biochemical methods for aneuploidy and open neural tube defect screening, it is important to accurately record maternal weight, ethnicity and diabetic status, as these characteristics have been shown to alter the levels of serum analytes [25,30,33,34]. Risk refinement algorithms for first- and second-trimester serum screening employ adjustments for maternal weight, ethnicity and diabetic status [33,34]. Several different adjustment algorithms have been proposed, though comparative clinical studies are lacking [33,34,35,36]. Obese women have lower levels of hCG, PAPP-A, uE3, AFP and DIA, presumably due to increased plasma volume and dilutional effects [34,37]. Given the known alteration patterns of these analytes in pregnancies affected by neural tube defects and trisomy, decreased analyte levels found in obese patients can result in false-negative results in open neural tube defect screening and false-positive results for trisomy 21 and trisomy 18 [33,34,35]. Weight-based adjustments in biochemical screening have improved the detection of neural tube defects, trisomy 18 and trisomy 21 [34,36,38,39,40]. Huang *et al.* found that a five pound weight discrepancy would change serum biochemical screen results from positive to negative (or from negative to positive) in up to 9.3% of women completing integrated prenatal screening and up to 2.9% of women completing first-trimester combined screening [40]. However, for those patients close to the risk cutoff thresholds, the number of changed results increased to 47.8%–54.8% and 9.5%–42.6%, respectively. These results underscore the importance of accurate maternal weight reporting when using biochemical aneuploidy screening methods.

### 2.3. Non-Invasive Prenatal Testing

Refinement of next generation DNA sequencing has allowed the adoption of non-invasive prenatal testing (NIPT) using cell-free fetal DNA (cffDNA) analysis as an effective means of aneuploidy detection in high-risk pregnancies. This technology relies on the ability to detect proportional increases in DNA derived from the aneuploid fetus against the much larger background of euploid maternal DNA. Large series have shown trisomy 21 and trisomy 18 detection rates with NIPT to be greater than 99% with a false positive rate less than 1% [41,42,43,44,45,46,47]. While NIPT appears superior to all other aneuploidy screening methods, its major drawback is the possibility of obtaining a non-informative result. 

The fetal fraction of cffDNA in maternal plasma is a critical determinant in the ability to obtain an informative and reliable result with NIPT. The fetal fraction remains relatively stable between 10 and 21 weeks of gestation, increasing by only 0.1% per week during this time period [48]. Fetal fractions less than 4% contain insufficient fetal DNA for counting-based technologies (shotgun and targeted massively parallel sequencing) and are unsuitable for analysis [42,45,49]. The strongest factor associated with an insufficient fetal fraction is increasing maternal weight [48]. Ashoor *et al.* showed that the estimated proportion of patients with a fetal fraction below 4% increased with maternal weight (0.7% at 60 kg, 7.1% at 100 kg, 51.1% at 160 kg) [50]. The proportion of patients with an insufficient fetal fraction in a 2013 study by Canick and colleagues was only 0.6% at a maternal weight of 60 kg, but increased to 3.3% at 100 kg and 23.7% at greater than 110 kg [51]. Wang *et al.* showed similar findings (0.8% at 60 kg, 6.1% at 100 kg, and 28.8% > 140 kg) [48]. Obese women with an insufficient fetal fraction on an initial blood draw may elect to have a repeat specimen drawn. An analyzable sample with a fetal fraction over 4% was obtained with repeat sampling in only 18% (≥140 kg) to 71% (<90 kg) of patients, with success varying inversely by maternal weight [48]. Ghanta *et al.* theorized that NIPT methods using a single nucleotide polymorphism (SNP)-based approach can provide accurate results with fetal fractions as low as 2%; thus, they may be preferable in obese patients with insufficient fetal fractions [49,52]. However, to our knowledge, such claims have not been validated in obese patients.

While the increased risk of an unreportable result in obese patients is concerning, the higher false negative rate in this population is of greater concern. The ability to distinguish between euploid and aneuploid pregnancies is diminished with decreasing fetal fraction. The proportion of DNA fragments mapped to the chromosome of interest (expressed as multiples of the median) overlaps more at lower fetal fractions, increasing the risk of a false negative result [51]. At fetal fractions above 10%, almost no false negatives are expected with counting-based technologies [51]. Median fetal fractions have been shown to decrease with increasing maternal weight from 14.2% at 60 kg to 9.2% at 90 kg [50,51]. At present, obesity-specific test statistics (sensitivity, specificity, false negative rate) are unknown and patients should be informed of the known limitations of NIPT in the setting of maternal obesity, especially those at the extremes of maternal weight.

### 2.4. Ultrasonographic Marker Screening (“Soft Markers”)

Ultrasound is used routinely to evaluate fetal anatomy, as well as to screen for “soft” markers to further define a woman’s aneuploidy risk and assist in genetic counseling. These soft markers include a thickened nuchal fold over 5 mm, echogenic intracardiac focus, echogenic bowel and renal pyelectasis [53]. Although these finding are often considered of limited utility when found in isolation after low-risk screening, when found in concert with mid-range or positive screening, they can help to refine risk [54]. 

The accuracy of using soft markers for screening relies on the consistent and appropriate identification of these markers. Just as increasing BMI can make obtaining accurate nuchal translucency measurements challenging, adequate visualization of soft markers can be similarly problematic. Tsai *et al.* examined the completion rates of ultrasound aneuploidy screening between normal, overweight and obese women [55]. In this study of approximately 5700 patients, completion rates differed significantly between groups and were inversely related to maternal BMI. In addition, the rate of screen positivity was lower in the obese group when compared to normal weight patients, as was the identification rate of commonly used markers for aneuploidy. In the subset of women undergoing invasive genetic testing, aneuploidy detection rates were significantly different, with 40% of the fetuses of normal or overweight patients having aneuploidy detected by ultrasound markers compared to 9% of fetuses among obese patients. Similarly, Aagaard-Tillery *et al.* found a significantly higher missed detection rate for several soft markers of aneuploidy, including increased nuchal fold, short humerus and echogenic focus in association with maternal obesity [53]. These data further suggest that, in the obese patient with a completed anatomic ultrasound, relying on the absence of soft markers of aneuploidy may not be as reassuring, since adequate visualization is limited.

### 2.5. Diagnostic Testing for Aneuploidy

Given the screening limitations for aneuploidy and anomaly in the obese population, invasive diagnostic testing may be recommended. The difficulties of ultrasound-guided procedures in obese gravida are well known; however, there are limited data regarding the risk of adverse outcomes following invasive testing in these patients. Harper *et al.* examined the rates of fetal loss following amniocentesis and chorionic villus sampling (CVS) in obese *versus* non-obese patients [56]. These data showed a statistically significant increase in the rate of fetal loss in the subgroup of patients with Class III obesity compared to non-obese women (2.7% *vs.* 1.3%, *p* = 0.03) [56]. In addition, a higher proportion of obese patients required multiple needle insertions than non-obese counterparts [56]. This study also showed a decreasing number of amniocenteses performed for abnormal serum screening with increasing BMI [56]. The authors postulated that this decrease may be due to fewer detected anomalies (to encourage diagnostic testing) or because healthcare providers discourage patients from undergoing the procedure when they perceive such a procedure will be difficult to perform [56].

## 3. Anomaly Detection

### 3.1. Early Anatomy Survey

It is the goal of modern obstetrics to attempt the prenatal diagnosis of anomalies at the earliest gestational age. First-trimester assessment of fetal anatomy, performed coincident with NT screening, has been advocated as a means to detect structural anomalies earlier in gestation. Though data on the effectiveness of this approach are somewhat conflicting, a recent meta-analysis found an overall first-trimester anomaly detection rate of 51% with higher detection rates for several major congenital anomalies, including gastroschisis/omphalocele, holoprosencephaly, hypoplastic left heart syndrome and tetralogy of Fallot [57]. It has been suggested that early anatomic evaluation using transvaginal sonography may compensate for the limited detection of structural anomalies using second-trimester transabdominal sonography in obese women; however, there are no obesity-specific data on the accuracy of first trimester sonography for the detection of fetal anomalies.

### 3.2. Limitations of Second-Trimester Ultrasound

Organizations, such as the American College of Obstetricians and Gynecologists (ACOG), the American Institute of Ultrasound in Medicine (AUIM) and the National Institute of Clinical Excellence (NICE) recommend fetal anatomic ultrasounds beginning at 18–20 weeks of gestation [58,59,60]. Given the known fetal complications among obese women, the fetal anatomic ultrasound plays a crucial role in prenatal care and the diagnosis of structural anomalies. The major technologic limitation of ultrasound in obese women is the distance from the skin to the intrauterine environment. The depth that the sound beam must travel is inversely proportional to image quality. Adipose tissue is echogenic, and excess abdominal fat results in the absorption of the sound beam. This absorption within the subcutaneous tissue leads to marked signal attenuation and decreased image clarity. Attenuation is further influenced by scatter, reflection and reverberation of the signal, which may compromise image quality [61,62].

### 3.3. Completion of Anatomic Surveys

Numerous studies have shown that obesity is associated with significant limitations in the ultrasound evaluation of fetal anatomy. Wolfe *et al.* showed a 14.5% decrease in the visualization of fetal anatomy among women with a BMI greater than the 90th percentile (36.2 kg/m^2^) [63]. Fuchs and colleagues reported significantly poorer image quality in obese women for all parameters, except abdominal circumference and spine [64]. In a study of approximately 10,000 women, including 2500 obese women, Dashe *et al.* reported a significant decrease in the number of pregnancies with completed fetal anatomic scans with increasing maternal BMI, 68% in normal weight women compared to 30% in women with a BMI > 40 [65]. In particular, the visualization of the midline face, four-chamber heart, spine and neuroanatomy was significantly decreased with obesity. In a similar cohort of 7000 women, Thornburg *et al.* showed that the completion rates for the spine, cardiac anatomy, profile and lips/nose were inversely associated with BMI class [66]. Similar difficulty in assessing cardiac and spinal anatomy has been reported elsewhere [62,65,67,68].

Poor visualization of the fetal heart is one of the main reasons for an incomplete anatomic survey. In a study of 372 women, Hendler *et al.* found that the rate of suboptimal visualization of fetal cardiac structures during the first ultrasound was significantly higher among women with Class III obesity compared to non-obese women (49.3% *vs.* 18.7%) [68]. There was also a gradual, but significant, increase in the rate of persistent poor visualization with increasing BMI at the follow-up examination [69]. Obese patients should be counseled on the risks of inadequate visualization of the fetal heart given the known increased odds of cardiac malformations in this population [21].

Thornburg *et al.* found that the completion rates of both basic and comprehensive anatomic surveys significantly declined in obese women: 79% of normal weight women had a completed basic scan compared to 49% in Class III obese women [66]. The number of ultrasound scans required to complete the anatomic surveys was also significantly higher among obese women [66]. A prospective study by Fuchs *et al.* assessed the feasibility of completing a second-trimester anatomic ultrasound in a single session among 223 obese women compared to 60 normal weight women [64]. They showed a non-statistically significant trend toward lower first-attempt completion rates in obese women (70.4%) compared to normal weight women (81.7%). Characteristics that predicted a successful second-trimester anatomic ultrasound in obese women were maternal abdominal wall thickness, an additional 10 min of ultrasound scanning time, fetus in the supine position and the experience of the ultrasonographer. Chung *et al.* reported a completion rate of 71.8% for first scans among women with Class III obesity compared to 83.6% among Class I obese women (*p* = 0.17) [67]. The study also reported that the total number of adequate ultrasound examinations significantly decreased among rising BMI categories (*p* < 0.05). Phatak and Ramsey further summarized unfavorable characteristics, including excessive operator strain, poorer image quality and a need for more time to complete the anatomic survey, that were significantly higher among obese women [60]. These considerations are of particular importance given that current ACOG and Internal Society of Ultrasound in Obstetrics and Gynecology (ISUOG) practice guidelines recommend follow-up exams if the first ultrasound is incomplete [70]. At the very least, obese patients should be counseled on the risks of inadequate visualization, the need for longer ultrasounds and the possible need for multiple follow-up examinations.

### 3.4. Performance in Anomaly Detection

In a secondary analysis of the National Institute of Child Health and Human Development (NICHD) First and Second Trimester Evaluation of Risk (FaSTER) trial (*N* = 8555 women), maternal BMI > 30 significantly decreased the likelihood of the detection of any congenital anomalies (*p* = 0.001) [53]. This trial also showed a higher false positive rate in the detection of cardiac anomalies in obese women compared to women with a BMI < 25 [53]. These findings have been replicated in other studies. In a large retrospective cohort of more than 10,000 standard ultrasound examinations, the detection of major anomalies decreased with increasing maternal BMI. For normal weight women, overweight women and those with Class I, Class II and Class III obesity, the detection rates were 66%, 49%, 48%, 42% and 25%, respectively [65]. A similar trend was seen among nearly 1100 targeted ultrasound examinations, ranging from 97% detection rates in normal BMI to 75% in Class III obese women [71]. A large population-based study of second trimester ultrasounds (*N* = 14,497) from Sweden showed that detection rates of congenital anomalies was 21% in obese women compared to 30% in normal weight women [72]. Using a population-based registrar of congenital anomalies, Best *et al.* reported a significantly lower detection rate of any congenital anomalies among obese women (adjusted OR 0.77, 95% CI 0.60–0.99) [73]. Although all patients should be aware of the potential for missed anomalies after ultrasound, this risk is further increased in obese patients.

### 3.5. Techniques to Improve Visualization

Many ultrasound and scanning techniques have been suggested as a way to improve imaging quality and diagnostic success in obese women [61,74,75]. The use of tissue harmonic imaging, speckle reduction filters, pre-processing and post-processing filters have been shown to increase the signal-to-noise ratio. Pannus retraction towards the patient’s head exposes the pubic bone and often times allows for improved visualization, due to the decreased tissue thickness in this area. This is also a useful technique during an amniocentesis [62]. Scanning the mid-abdomen above the pannus, using the acoustic window through the umbilicus, bladder filling and placing the patient in the lateral decubitus position may further improve visualization [61,74,75]. The latter technique allows a subcostal approach to the uterus by shifting the abdomen toward the table and results in greater pressure on the uterus, which may produce greater image clarity. Similarly, Benacerraf reported the successful use of the Sims position, where the abdomen and panniculus are shifted onto the ultrasound table, while the patient lies prone [61]. The patient’s upper leg is then flexed, while the lower leg is extended. The ultrasound scan is performed through the flank, where the thickness of adipose tissue is decreased. A transvaginal approach may also improve visualization by placing the transducer closer to the fetal anatomy [67]. Several studies have reported that the probability of completing or obtaining an adequate ultrasound scan in obese women improves with sonographer experience [64,67,69].

## 4. Conclusions

The obesity epidemic is generating a global impact on obstetrical practice, resulting in more complicated pregnancies for both the mother and fetus. Maternal obesity influences the diagnostic abilities of available screening modalities and has consistently been shown to decrease the performance of both first- and second-trimester serum and ultrasound screenings. Given the increased risks associated with maternal BMI, the screening limitations are concerning. Preconception counseling and weight loss should be encouraged. Furthermore, communication and expectations regarding the limitations for aneuploidy and anomaly screening among these women should be initiated early in care. Raising awareness of these risks allows for appropriate patient counseling and improved pregnancy management.
